# A study of generalization and compatibility performance of 3D U-Net segmentation on multiple heterogeneous liver CT datasets

**DOI:** 10.1186/s12880-021-00708-y

**Published:** 2021-11-24

**Authors:** Baochun He, Dalong Yin, Xiaoxia Chen, Huoling Luo, Deqiang Xiao, Mu He, Guisheng Wang, Chihua Fang, Lianxin Liu, Fucang Jia

**Affiliations:** 1 Shenzhen Institute of Advanced Technology, Chinese Academy of Sciences, Shenzhen, China; 2 Shenzhen College of Advanced Technology, University of Chinese Academy of Sciences, Shenzhen, China; 3 Department of Hepatobiliary Surgery, The First Affiliated Hospital, Harbin Medical University, Harbin, China; 4 Department of Hepatobiliary Surgery, The First Affiliated Hospital, University of Science and Technology of China, Hefei, China; 5 Department of Radiology, The Third Medical Center, General Hospital of PLA, Beijing, China; 6 First Hepatobiliary Surgery, Zhujiang Hospital, Southern Medical University, Guangzhou, China; 7 Pazhou Lab, Guangzhou, China

**Keywords:** Liver segmentation, U-Net, Dataset-wise convolution, Generalization

## Abstract

**Background:**

Most existing algorithms have been focused on the segmentation from several public Liver CT datasets scanned regularly (no pneumoperitoneum and horizontal supine position). This study primarily segmented datasets with unconventional liver shapes and intensities deduced by contrast phases, irregular scanning conditions, different scanning objects of pigs and patients with large pathological tumors, which formed the multiple heterogeneity of datasets used in this study.

**Methods:**

The multiple heterogeneous datasets used in this paper includes: (1) One public contrast-enhanced CT dataset and one public non-contrast CT dataset; (2) A contrast-enhanced dataset that has abnormal liver shape with very long left liver lobes and large-sized liver tumors with abnormal presets deduced by microvascular invasion; (3) One artificial pneumoperitoneum dataset under the pneumoperitoneum and three scanning profiles (horizontal/left/right recumbent position); (4) Two porcine datasets of Bama type and domestic type that contains pneumoperitoneum cases but with large anatomy discrepancy with humans. The study aimed to investigate the segmentation performances of 3D U-Net in: (1) generalization ability between multiple heterogeneous datasets by cross-testing experiments; (2) the compatibility when hybrid training all datasets in different sampling and encoder layer sharing schema. We further investigated the compatibility of encoder level by setting separate level for each dataset (i.e., dataset-wise convolutions) while sharing the decoder.

**Results:**

Model trained on different datasets has different segmentation performance. The prediction accuracy between LiTS dataset and Zhujiang dataset was about 0.955 and 0.958 which shows their good generalization ability due to that they were all contrast-enhanced clinical patient datasets scanned regularly. For the datasets scanned under pneumoperitoneum, their corresponding datasets scanned without pneumoperitoneum showed good generalization ability. Dataset-wise convolution module in high-level can improve the dataset unbalance problem. The experimental results will facilitate researchers making solutions when segmenting those special datasets.

**Conclusions:**

(1) Regularly scanned datasets is well generalized to irregularly ones. (2) The hybrid training is beneficial but the dataset imbalance problem always exits due to the multi-domain homogeneity. The higher levels encoded more domain specific information than lower levels and thus were less compatible in terms of our datasets.

## Background

Medical image segmentation is very important as it is the prerequisite in feature extraction, surgery planning, and image guided interventions. Liver is among the biggest soft organs and CT is the primary modality in clinical liver imaging. For three decades, many methods have been proposed to achieve automatic, robust, and accurate liver segmentation to alleviate the manual slice-to-slice delineation work [1, 2]. In contrast to traditional methods such as atlas-based methods [3, 4], graphical models [5, 6], and deformable models with shallow machine learning methods [7, 8], which achieved limited accuracy with a lot of user-specified parameters and poor generalization performances, the revolutionary deep learning based segmentation was fully automatic and have shown great success in medical image segmentation [9–13]. Recently liver segmentation methods based on the popular U-net model [11–13] was proposed and can reach the highest Dice similarity coefficient (DSC) value of 0.985. It seems that liver segmentation problem has been addressed so far.

However, most methods improved the segmentation from those public datasets such as 3Dircadb1 [14], Sliver07 [15] and LiTS [16]. Those datasets were all scanned regularly (no pneumoperitoneum and horizontal supine position) from patients. Except Sliver07, both 3Dircadb1 and LiTS have tumors. LiTS was the largest public liver tumor segmentation dataset with diversity in size, numbers, and medical centers. The liver tumors in LiTS have lower intensity than liver and maximum size of 349 cm^3^ (25% of the whole liver). Instance with small-size tumors is more difficult for liver tumor segmentation [11] but easier for binary segmentation of the whole liver because large various lesion presets [17] may make the liver shape and intensity out of distribution [18, 19].

The datasets in this study were quite different from existing public datasets in both liver shape and intensity. For instances, the Zhujiang dataset scanned from microvascular invasion [20–22] patients exhibit high-intensity large tumors and abnormal liver shape with very long left liver lobes. Our previous study analyzed the 3Dircadb1 dataset that contains cases like long liver lobe and tumor growing outside of the liver [18]. Its segmentation accuracy was worse than Sliver07 [18] and LiTS even for the SOTA method modified-UNet [13] due to small samples (twenty) and abnormal cases. The APP (artificial pneumoperitoneum) datasets has large deformation and porcine datasets have anatomy discrepancy with humans. However, segmentation of these datasets was required in clinical [23] and animal experiments [24] like pneumoperitoneum deduced anatomical changes [23] and its deformation registration [25] or modeling [26–29] but gained few attentions. Our previous study [23] show that pneumoperitoneum CT favored the augmentation reality in laparoscopic liver resection. Unfortunately, we only traced few literatures related to the segmentation of animal CT such as mouse multi-organ segmentation [30].

Researchers have few literatures to refer facing challenges of lacking training samples for these segmentation tasks in a short time. Developing segmentation algorithms consumed time as radiologists are often busy. A fully automatic and robust tool is needed for rapid training and prediction. U-Net [31] has been established a universal and robust medical image segmentation method. Schoppe et al. [30] developed an AIMOS processing pipeline based on U-Net for multi-organ segmentation in whole-body mouse CT scans and concluded that deep learning method can address the issue of human bias of expert annotations. Excitingly, a similar but fully automatic pipeline named nnU-Net (no new U-Net) proposed by Isensee et al. [12] has achieved satisfied results on 53 segmentation tasks. They made it a public tool accessible to a broad audience by requiring neither expert knowledge nor computing resources beyond standard network training [31]. Zhou et al. [32] proved that the 3D U-Net performed better than 2D in liver segmentation. The 2D U-Net lose 3D information and predicted a discontinuous area requiring more manual correction. Hence, we choose 3D U-Net version in nnU-Net and the network architecture novelty is not the focus in this paper. The most important problem is lacking training samples.

The first promising idea was that whether we can exploit the related public labeled dataset for the new task? However, in contrast to humans, deep learning model may have poor generalization ability facing domain-shifted task [33, 34]. In terms of our datasets, the homogeneity was deduced by contrast phases, pathological type, population and shape deformation by irregular scanning profile with pneumoperitoneum. What’s the feasibility extent of using the dataset scanned regularly to predict samples scanned irregularly, or using the patients to predict animal pigs? Hence, the first question is what’s the generalization relationships between the special datasets used in our study? The unsupervised method made us step first to annotate a new dataset, avoiding completely manual delineation especially for those large organs. Thus, cross-dataset testing results will facilitate researchers to choose most related datasets for unsupervised segmentation or for data augmentation.

Secondly, whether a hybrid training of all datasets (multi-domain learning) can generate a more generalized model if direct prediction and transfer learning failed for some datasets like porcine dataset? For humans, diverse datasets in terms of one task contribute to learning the essence of the object. Big data is particularly important for the data-driven deep learning model. Mårtensson et al. [33] also suggests that more heterogeneous MRI training data makes the model generalize better. However, if those datasets have strong homogeneity, the compatibility of U-Net is not clear [35, 36]. It is hard to avoid the dataset unbalance problem because our datasets have different distributions and sample sizes. Our previous study [37] found that the hybrid training of non-contrast CT data with the same number of contrast CT data can improve segmentation performance of non-contrast dataset. But the segmentation accuracy decreased when increasing the proportion of contrast data. The sample size problem can be alleviated by sampling strategy while the heterogeneity problem is the source reason. In terms of multi-domain segmentation, researchers proposed a variety of methods to improve the representative ability of U-Net on MRI datasets. Leonardo et al. [38] incorporated a SE (Squeeze and Excitation) block in all levels of the encoder. Liu et al. [39] used separate decoder for each dataset. All of those works considered that the encoder contain more domain-specific information. However, which level encodes more domain specific information is not investigated. The results contribute to designing networks that can preserve the generative features while separately learn or fine-tune the discriminative features in multi domains [39, 41] and domain adaption learning [40].

Existing studies investigated the characteristics of the learning features in each encoder level by visualizing features [18] from encoder, or fine-tuning from different levels [42, 43]. Different from previous study, we proposed a dataset-wise convolution module on each level of the encoder while fixing the decoder to estimate the encoder levels’ compatibility in multi-datasets learning. Moreover, the dataset-wise convolution module can alleviate the dataset-imbalance problem by replacing fewer compatible levels that encode more domain-specific information.

In summary, we will investigate the segmentation performances of 3D U-Net on multiple diverse liver CT datasets. The results will provide some references when we making labels from scratch. The contributions of this paper are:

1. The segmentation performances of supervised U-Net segmentation model on multiple diverse liver CT datasets were investigated in two aspects: (1): inter-dataset generalization performances by cross-testing experiments; (2) the compatibility by hybrid training all datasets in different sampling and encoder layer sharing schema. We used a novel dataset-wise convolution module (DCM) to explore the compatibility of each level in the encoder. The dataset imbalance problem can be alleviated by replacing those fewer compatible levels with DCM.
2. From the dataset view, this is the first publication of using 3D U-Net model for porcine liver CT segmentation, and also the first related to the segmentation of liver CT segmentation under pneumoperitoneum pressure with different scanning protocols.

The remaining structure of the paper was as follows. Method section describes the details about method and datasets. The experimental settings and results grouped by the two aspects of segmentation performances were given in Experiments section and Results section, followed by discussions and the conclusions.

## Methods

### Network architecture

**Fig. 1:**
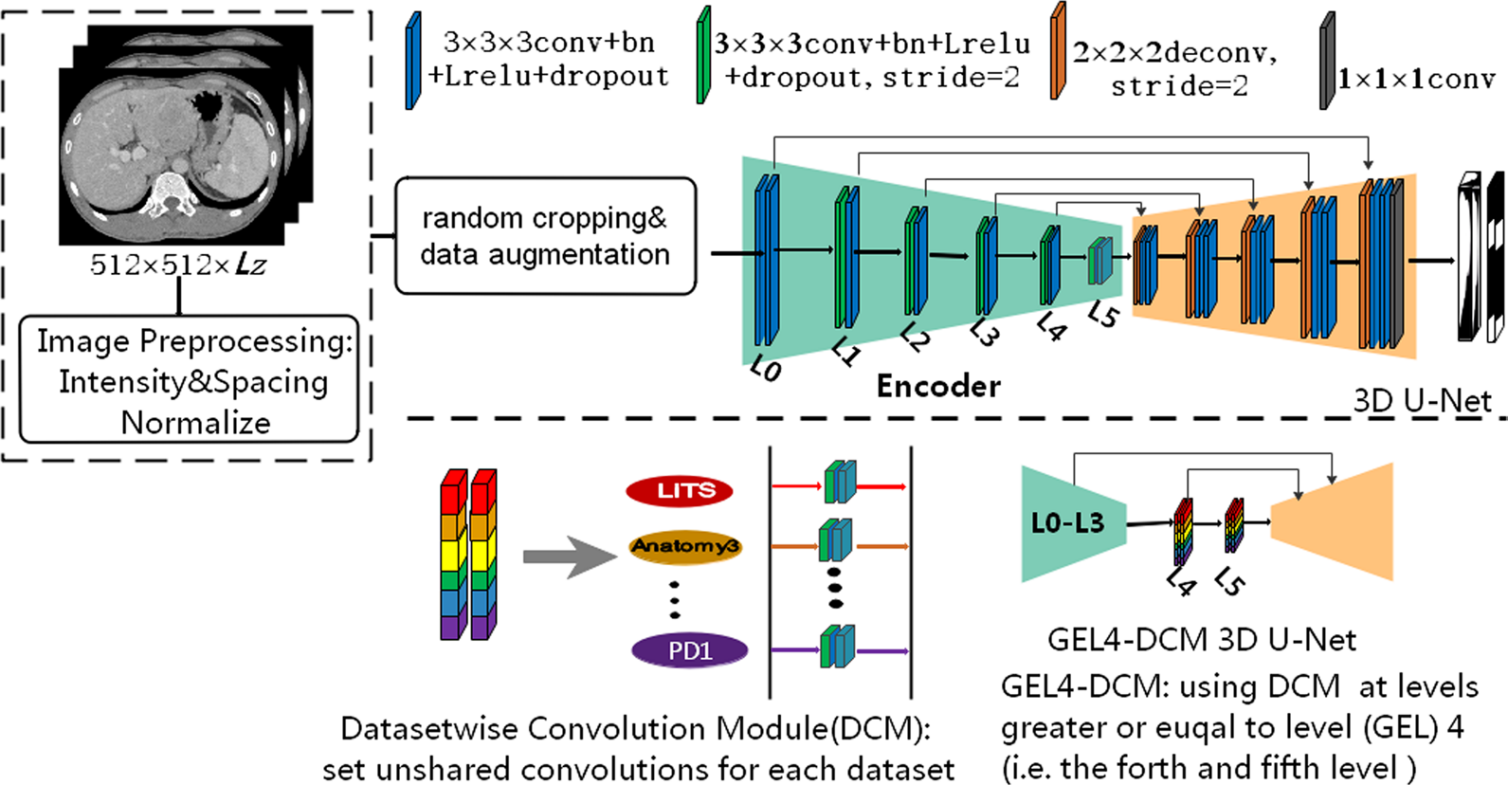
The pipeline of the method and the segmentation networks used in this study. The proposed dataset-wise convolution module (DCM) sets separate level convolutions for the eleven datasets. Convolution layers in the encoder was leveled by the size of their output features maps and numbered by down-sampling time. GEL*N*_DCM 3D U-Net set DCM at those levels greater or equal to Level (GEL) N (N ranges from zero to five) in the encoder of 3D U-Net

The 3D network used in this study was a conventional 3D U-Net shown in Fig. 1. The initial feature map and batch size was set as 32 and 2, respectively. The number of feature maps doubled but cannot exceed 320 and the size down-sampled by half after each stride convolution. We add the combination of batch normalization, leakyReLU, and dropout after each convolution with kernel 3. The up-sample method adopted transposed convolution with both kernel and stride size of 2. Finally, convolution with 1 kernel size was used to output the target number of feature maps. Convolution layers in the encoder were leveled by the size of their output features maps and then numbered by the down-sampling times. For an instance, the first two convolutions with output size of 128 × 128 × 128 and down-sampled times of zero were named Level 0.

To investigate the generalization performances of the level features in the encoder, we proposed dataset-wise convolution module that sets separate level convolutions for the eleven datasets by fixing the decoder. The GELN_DCM 3D U-Net using dataset-wise convolution module (DCM) at all encoder levels with their level number greater than or equal to N (GELN) in the 3D U-Net. For an instance the GEL4_DCM network replaces the original convolutions with dataset-wise convolutions at Level 4 and Level 5. We do not use dataset-wise convolution at only level N for GELN_DCM 3D U-Net based on the phenomena that the higher levels were less compatible than lower levels in terms of our datasets. For GEL4_DCM 3D U-Net, if we only use DCM in level 4 but shared in level 5, the effeteness of DCM may not be obvious compared to fully shared schema as the less compatible level 5 may still learn the distribution of some dataset and neglect the unbalanced dataset. Then the generalization performance of the n’th level was estimated by comparing GELN_DCM network with GELM_DCM (M is greater than N).

### Learning parameters and training details

Our training methods were based on nnU-Net. Data augmentation was implemented using batch generators with transform types of scale, flip, rotation, elastic deformation, and gamma correction. The loss was the sum of Dice and cross entropy. The optimizer was Adam with learning rate initially set as 3 × 10^−4^ and updated using ReduceLROnPlateau scheduler with patience of 30 epochs. The nnU-Net adopted the automatically training stop conditions that the training losses did not improve by at least 5 × 10^−3^ within the last 60 epochs and the learning rate was smaller than 10^−6^. Finally, the number of training epochs was less than 400 with 250 iterations each. The training consumption of GPU memory was about 12 GB, and the time required for the whole training was about 33 h.

### Data preparation

**Fig. 2:**
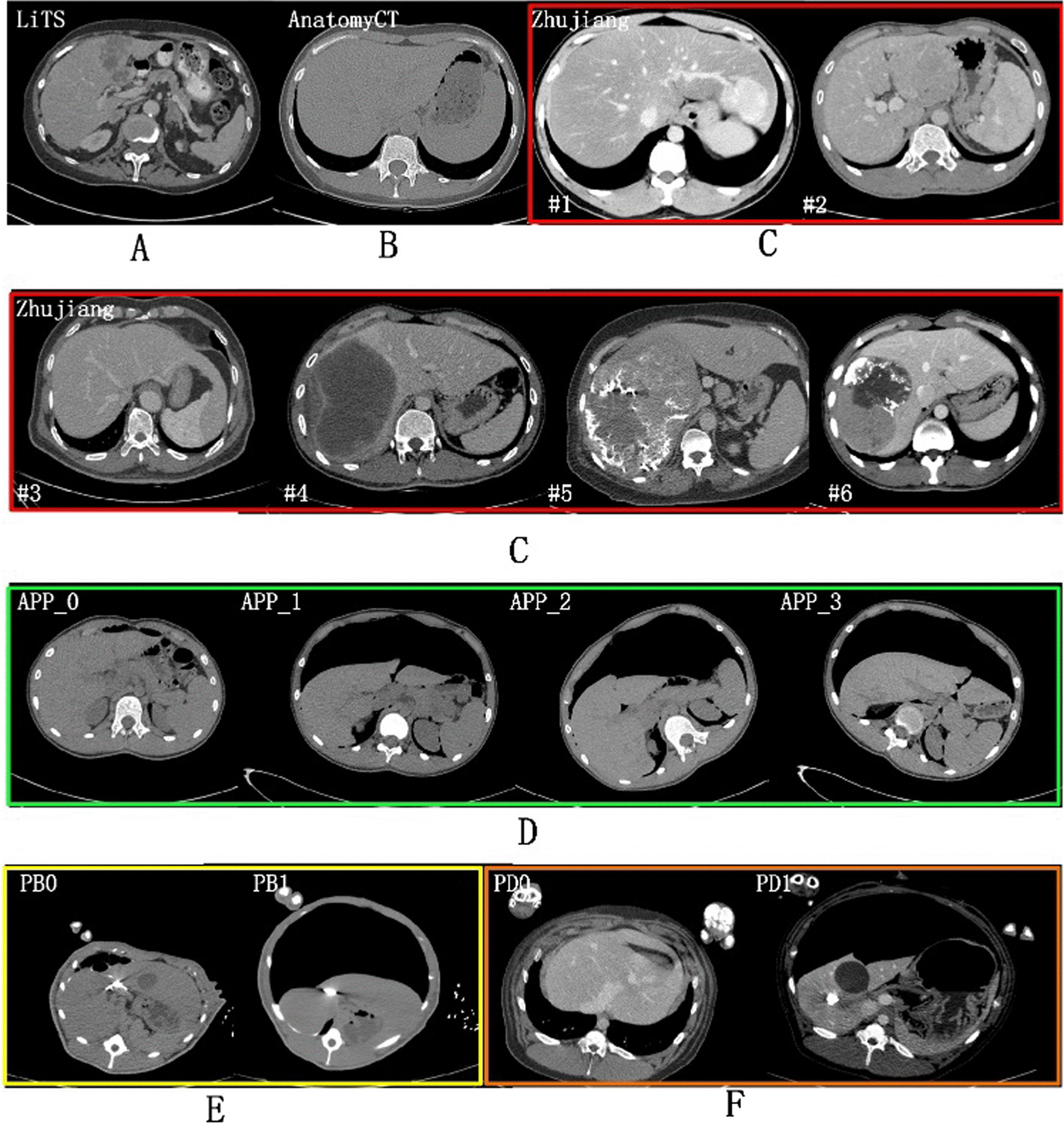
Multiple liver CT datasets in different scanning conditions—**A** public contrast-enhanced CT liver tumor dataset from five developed countries in LiTS Challenge, **B** public non-contrast CT normal liver segmentation dataset in Anatomy3 Challenge, **C** clinical patients with long left liver lobes (case #1 and #3) and large and intensity-varied (low or high) liver tumor changes from Zhujiang Hospital in China, **D** non-contrast CT dataset from real patients scanned regularly (APP_0) and irregularly (APP_1-3) with different scanning profiles under artificial pneumoperitoneum (APP) pressure, **E** non-contrast Bama Pig (PB) CT dataset and **F** contrast-enhanced domestic pig (PD) CT with (PB1 & PD1) or without (PB0 & PD0) pneumoperitoneum pressure

- Dataset: This study used six datasets listed as follows:
  1. LiTS: This public dataset consisted of 130 abdomen contrast enhanced CT volumes scanned from multiple clinical sites in five developed countries [44]. The dataset was aimed for liver and liver tumor segmentation in the challenge of ISBI2017-LiTS and MICCAI2018- medical segmentation decathlon. In our study we will focus on the whole liver containing tumor segmentation.
  2. Anatomy3: This public dataset came from the multi-organ segmentation challenge hold with ISBI 2015 [45]. It contained 20 non-contrast enhanced abdomen CT scans from real patient.
  3. Zhujiang: The dataset mainly comes from one of our research group study about radiomic feature-based predicting model for microvascular invasion in patients with HCC [20]. This dataset included 164 contrast enhanced CT scans from clinical treatment of real patients diagnosed with hepatic cysts, cancer, hepatocirrhosis provided by Zhujiang Hospital affiliated with Southern Medical University. The Zhujiang dataset has abnormal liver shape with very long left liver lobes (Fig. 2. case #3 and #1). Such samples occupied 16.5%. It also has abnormal liver tumors due to enhanced arterial phase ((Fig. 2. case #1 has tumor intensity higher than liver), microvascular invasion (case #5 and case #6), very large tumors and tumor growing outside of liver (Fig. 2. case #2).
  4. APP: (artificial pneumoperitoneum): This dataset came from 58 patients under the abdominal wall adhesion check through scanning CT images with pneumoperitoneum pressure and different profiles. The datasets were provided by the Third Medical Center, General Hospital of PLA. We named those datasets as APP (0–3) for each scanning profile. APP_0 means the regular supine profile without pneumoperitoneum. APP (1–3) were for the other three profiles: supine/left and right recumbent position under pneumoperitoneum. Most patients have all the scan positions.
  5. Porcine-B and Porcine-D: Both datasets came from our previous animal study about porcine anatomy change on pneumoperitoneum pressure conducted in the First Affiliated Hospital of Harbin Medical University in the years 2017 (Porcine-B) and 2018 (Porcine-D) respectively [23], which were approved by the Institutional Animal Care and Use Committee of Harbin Medical University. All the porcines came from the Heilongjiang Qing'an Jubao Pig Breeding Farm (Qing'an, Heilongjiang, China). Porcine-A contains 10 abdominal non contrast enhanced CT scanned from Bama pigs weighted 17–35 kg before and after 13 mmHg pneumoperitoneum. Porcine-B contains 8 contrast enhanced male domestic pigs weighted 25–49 kg and scanned also with and without 13 mmHg pneumoperitoneum. The porcines were sacrificed by administration of potassium chloride after experiments.
- Ground truth: For Zhujiang dataset the liver was segmented by homemade segmentation software [18] and manually corrected [20, 46] by experts. For APP dataset, we first used automatic segmentation based on the model trained with public dataset and then manually corrected by two experienced experts in abdomen disease treatment. Then a new model was trained for second automatic segmentation. Finally, all samples were manually corrected by experts. The porcine datasets were very difficult to annotate due to the poor data quality. In order to ensure that the surface of the sketch is smooth and accurate, it is necessary to repeatedly sketch out the uncertain areas and confirmed by generating the 3D surface model. It takes five hours each to accurately annotate a porcine label. The process of making liver segmentation labels inspired us to do the generalization ability study of those diverse liver CT datasets.
- Image Pre-processing*:* The image pre-processing included cropping, down-sampling, and intensity normalization. We borrowed the spacing and intensity normalization method from the nnU-Net [12]. First all images were cropped according to the bottom slice and upper slice of liver in z-axis, we relaxed the crop range by 60 mm in each side. Let (*L*_*x*_*, L*_*y*_*, L*_*z*_) denoted the size of original image, ${L}_{z}^{0}$ and ${L}_{z}^{1}$ represented the first and the final slice number of liver, respectively. Then images were cropped with the range of
  $$\left(\left(0, {L}_{x}\right),\left(0, {L}_{y}\right),\left(\mathrm{max}(0,{L}_{z}^{0}-60/{S}_{z}), \mathrm{min}({L}_{z},{L}_{z}^{1}+0/{S}_{z})\right)\right)$$
  to include enough context. Here ${S}_{z}$ was the spacing of image in z-axis. Second, all cropped images were first resampled as 128 × 128 × 128, then the media spacing was selected as the target spacing for resampling the original cropped images. By doing this, most images were resampled close to the designed input size of the 3D U-Net. In the final step, all down-sampled images were normalized by a z-score normalization based on the mean and standard deviation of target organ intensities in each dataset. Finally, preprocessed images were fed to the 3D U-Net model after random cropping and data augmentation.

## Experiments

To investigate the generalization relationship intra and inter the eleven datasets, we set a series of experiments with multiple training configurations listed as follows:

1. Inter-datasets cross-testing: In this experiment, for each dataset we train a model with its all samples and tested on all the other datasets.
2. Baseline: We compared two fully supervised method in two-fold and five-fold cross-testing which were named Fold_2 and Fold_5 respectively. The dividing of two-fold and five-fold was preceded by first sorting filenames. The two-fold cross-testing was used for all experiments. Thus Fold_2 was also set as a baseline.
3. Pre-train with LiTS: We also utilize pre-train model from LiTS dataset to investigate whether those various datasets can benefit from pre-training by doing paired t-test with the Fold_2 result. The learning rate of the pre-training is set very small as 1e-6 to constrain the over-tuning effect.
4. Hybrid training of all datasets: In this experiment, the two-fold cross-testing was performed like this: at first each dataset was divided by two folds, and then one of their corresponding folds was mixed for training and the other fold was for testing. We adopt three sampling strategies at each iteration:

***(a) Dataset-order sampling (DOS)***: random select a batch of samples from one dataset, and the selection was conducted in the order of the dataset listed in Table 1 for dataset balance.

**Table 1:** Details of the datasets used in this study

DataSet		Modality	Scan profile	No	PP
LiTS		CTce	Regular supine	130	N
Anatomy3		CT	Regular supine	20	N
Zhujiang		CTce	Regular supine	164	N
APP_	0	CT	Regular supine	34	N
	1	CT	Regular supine	56	Y
	2	CT	Left recumbent	49	Y
	3	CT	Right recumbent	51	Y
Porcine-Bama	B0	CT	Regular supine	10	N
	B1	CT	Regular supine	10	Y
Porcine-Domestic	D0	CTce	Regular supine	8	N
	D1	CTce	Regular supine	8	Y

***(b) Random sampling from one dataset (RSD):*** random select one sample from all mixture training samples and then choose a batch of samples from the dataset of the selected sample.

***(c) Random sampling (RS):*** random select a batch of samples from all mixture training samples. We compared GELN_DCM where N set from zero to five in DOS strategy to investigate the level generalization ability.

## Results

### Cross-datasets testing results

Table 2 gives the results of cross-testing where S and T represented training and testing dataset respectively. In Table 2, the prediction accuracy between LiTS dataset and Zhujiang dataset was about 0.955 and 0.958 which shows their good generalization ability due to that they were all contrast-enhanced clinical patient datasets scanned regularly. Although LiTS and Zhujiang have same magnitude of training samples, the generalization ability of LiTS is much higher than Zhujiang when predicting other datasets. Especially when predicting AnatomyCT dataset, LiTS and Zhujiang achieved accuracy of 0.907 and 0.633, respectively. This is because the Zhujiang dataset contains very large tumors with high intensity, resulting much over-segmentation in supine and muscle area. The LiTS samples were consistent in the presenting of tumors with low intensity and small tumors mostly.

**Table 2:** Cross-testing results between multiple datasets where S denotes source dataset and T denotes testing dataset

S	T										
	LiTS	Anatomy CT	Zhujiang	App-0	App-1	App-2	App-3	PB0	PB1	PD0	PD1
LiTS	–	0.907	**0.958**	0.943	0.937	0.909	0.889	0.795	0.732	0.860	0.832
AnatomyCT	0.661	–	0.630	0.858	0.857	0.882	0.861	0.797	0.783	0.815	0.781
Zhujiang	**0.955**	0.633	–	0.925	0.908	0.892	0.805	0.650	0.555	0.827	0.728
App_0	0.926	0.881	0.905	–	**0.954**	0.935	0.928	0.824	0.767	0.903	0.880
App_1	0.793	0.866	0.740	**0.954**	–	**0.943**	**0.949**	0.768	0.835	0.866	0.855
App_2	0.795	0.821	0.764	0.938	0.950	–	0.937	0.820	0.813	0.892	0.881
App_3	0.662	0.880	0.591	0.844	**0.957**	0.922	–	0.754	0.828	0.831	0.843
PB0	0.789	0.788	0.776	0.847	0.842	0.878	0.847	–	**0.830**	0.886	0.849
PB1	0.677	0.714	0.644	0.733	0.853	0.830	0.854	**0.879**	–	0.872	0.888
PD0	0.830	0.711	0.816	0.884	0.873	0.883	0.851	0.722	0.636	–	**0.932**
PD1	0.581	0.787	0.566	0.730	0.848	0.853	0.837	0.457	0.454	**0.921**	–

The bold denotes the best segmentation result of T from S

**Fig. 3:**
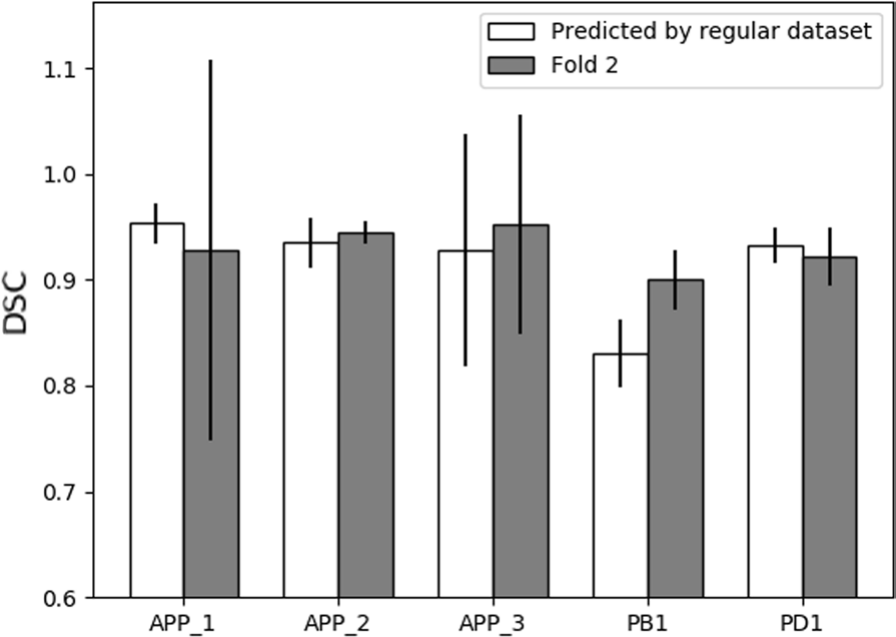
Bar chart of comparison results measured with DSC for datasets scanned under pneumoperitoneum which are predicted by their corresponding dataset scanned regularly (without pneumoperitoneum) and two-fold model respectively. Dataset scanned without pneumoperitoneum showed good generalization ability

For the datasets scanned under pneumoperitoneum such as APP-1, 2, 3, PB1, and PD1, their corresponding datasets APP_0, PB0, and PD0 scanned without pneumoperitoneum showed good generalization ability. To further testify this, we compared the datasets predicted by corresponding regular scanned datasets with two-fold testing results seen as Fig. 3. The average Dice value was higher when predicted by regularly scanned datasets than fully supervised results for the datasets APP_1 and PD1. In addition, the LiTS can predict APP_1 with DSC of 0.937. But for the other scan profile APP_2 & 3, the prediction accuracy was only 0.909 and 0.889. In inverse direction APP_1–3 predict LiTS with only 0.793, 0.795, and 0.662, much lower than APP_0 of 0.925 even with more training samples. The APP_3 dataset was most heterogeneous as the liver largely deformed to the left side (liver was positioned on the right side of the body) under left recumbent position.

The DSC cross testing between real patient datasets and porcine datasets was generally around 0.8, which means weak generalization ability between real patient and porcine liver segmentation as they anatomically differ too much.

It is obvious that the larger appearance gap between the datasets, the worse generalization ability. The factor includes scan object type, pathological type, scan regularly or not and contrast-phases. For each dataset, the best perdition result (bolded) was yielded by its closest dataset. LiTS and Zhujiang were best prediction dataset to each other as they have same factors in scan object, contrast enhanced and regular scan. For APP dataset and porcine dataset, the sub-datasets were best-prediction dataset as they contain the same patients or pigs.

### Dataset-wise convolution module in high-level can improve the dataset unbalance problem

**Fig. 4:**
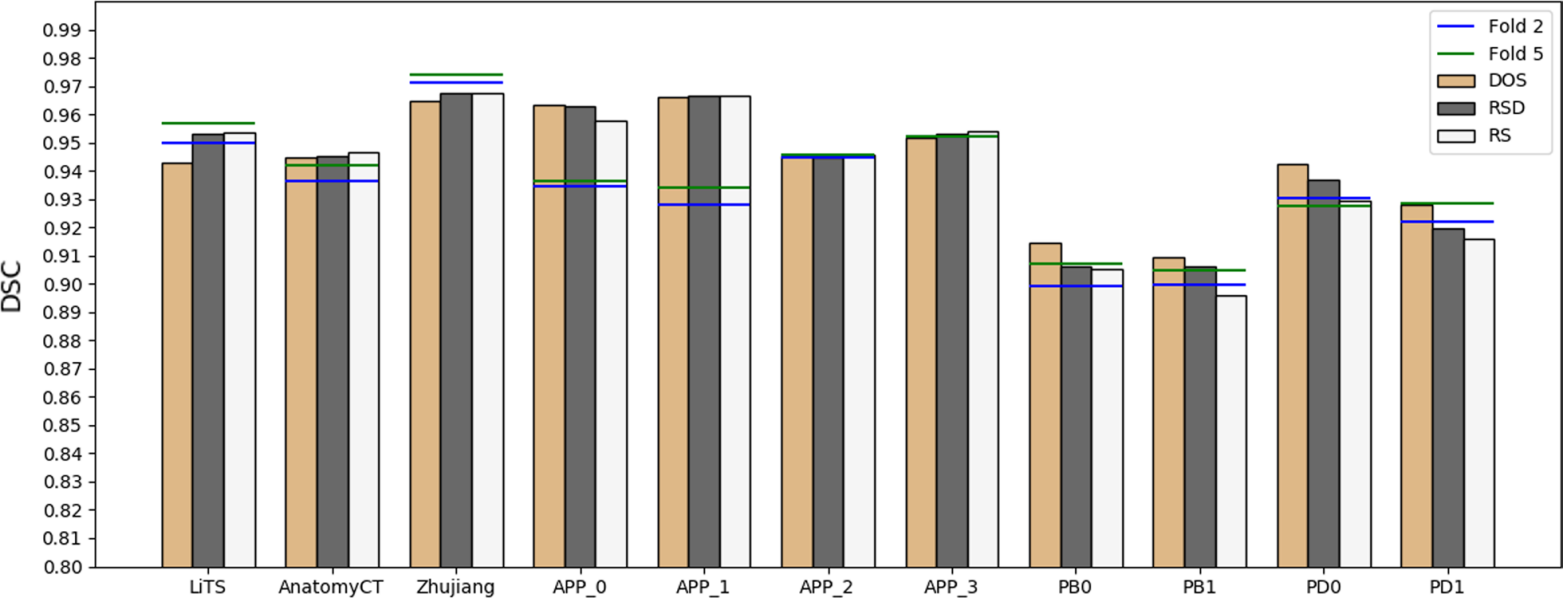
Bar charts of comparison results measured with DSC for eleven datasets grouped by different sampling strategies when training all datasets together by two-fold (the non-hybrid training schema fold 2 and fold 5 were used as baseline). The dataset-balance extent of sample strategy decreased from DOS > RSD > RS. Most datasets benefit from the hybrid training except the unbalanced dataset. LiTS and Porcine dataset was unbalanced dataset in DOS and RS strategy respectively. Zhujiang dataset cannot benefit from hybrid training in any sample strategy

Figure 4 compared different sampling strategies when hybrid training all those datasets in two-fold manner. The unbalance problem always exists whatever sample strategy used, suggesting some degree of incompatibility of those diverse datasets. Figure 4 showed that using DOS sample schema, all datasets except LiTS and Zhujiang benefit from the hybrid training which acquired higher DSC than the two-fold training baseline (denoted as horizontal blue line), especially for those datasets have few samples. With the increasing randomness from DOS to RSD and further to the complete random sampling of RS, the Dice value decreased for porcine datasets with few training labels while increased for human datasets.

**Fig. 5:**
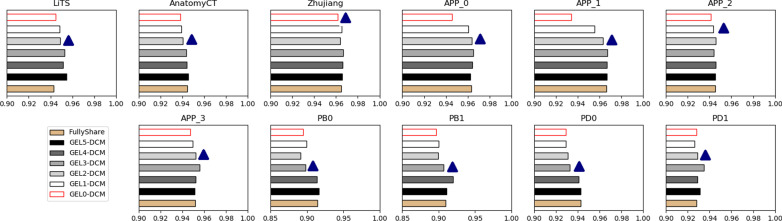
Bar charts of comparison results measured with DSC for eleven datasets tested by hybrid-training models with different encoder layer sharing schema. FullyShare was another name of the DOS result in Fig. 4. GELN_DCM denotes segmentation from GELN_DCM 3D U-Net in Fig. 2. The blue triangle denotes an obvious accuracy-decreased stagnation level, which suggested that the stagnation level and the lower levels should be shared and thus were more compatible. The GEL5-DCM can improve the unbalanced datasets’ accuracy while not reduce others’, which suggested that the final level of the encoder was the least compatible

Figure 5 further investigated the problem from viewer of compatibility of different leveled features in the encoder. Overall, the low-level features have better compatibility than high-level features. The compatibility for the fifth level was uniformly worse for all datasets. Compared with fully-share schema, GEL5_DCM network improved the segmentation performance for both the unbalanced dataset (LiTS) and the inclined datasets (APP & porcine) while not decreasing others. The close result of GEL4_DCM and GEL5_DCM means the compatibility of level 4 is not obviously good. Conversely, the most general and obvious decrease of GEL0_DCM compared to the GEL1_DCM schema means the Level 0 should be shared and thus was most compatible. The compatibility for other levels differs in dataset. For dataset LiTS & AnatomyCT & APP (0, 1, 3) & PB1 and PD1, an obvious decreasing start from Level 2 which means the Level (0–2) should be shared and thus were more compatible. For dataset PB0 & PB1 & PD0, it started from Level 3. The decreasing started from Level_0 and Level_1 respectively for dataset Zhujiang and APP_2. Thus, the sharing of lower level (0–3) convolutions is beneficial and thus was more generalized. Overall, the dataset unbalance problem can be addressed by setting the proposed dataset-wise convolution module simply only in Level 5.

Table 3 gives ablation results in the Hausdorff distance results by our proposed DCM hybrid training method with dataset order sampling strategy. Compared with non-hybrid-training 3D U-Net, the hybrid training using DOS (3D U-Net + DOS) cannot solve data unbalance problem. Datasets like LiTS, Zhujiang, PD1 produced worse results when using hybrid training. But by adding DCM in the fifth level of the encoder, our method produced smaller average Hausdorff distance with much smaller standard division then non-hybrid training for all datasets.

**Table 3:** Ablation results of the hybrid training schema using DCM at the last level of the encoder with DOS sample strategy measured with 95% Hausdorff distance for eleven datasets

	LiTS	Anatomy CT	Zhujiang	App-0	App-1	App-2	App-3	PB0	PB1	PD0	PD1
3D U-Net (Non-hybrid Fold_2)	10.274 ± 10.011	9.672 ± 5.656	9.027 ± 8.217	9.567 ± 17.000	9.298 ± 14.819	7.496 ± 4.789	7.488 ± 6.433	13.942 ± 5.613	12.733 ± 4.503	16.015 ± 5.138	11.900 ± 4.582
3D U-Net + DOS	11.334 ± 9.217	7.553 ± 2.120	10.055 ± 8.587	6.167 ± 4.093	5.354 ± 2.459	6.909 ± 4.145	6.707 ± 5.913	12.721 ± 3.643	12.035 ± 5.114	10.534 ± 3.541	17.557 ± 11.253
3D U-Net + GEL5-DCM + DOS	10.249 ± 6.400	8.786 ± 2.992	10.035 ± 8.779	5.519 ± 4.017	6.353 ± 2.813	6..410 ± 3.989	7.193 ± 7.354	12.688 ± 5.840	12.991 ± 5.371	10.006 ± 4.359	10.477 ± 4.826

**Fig. 6:**
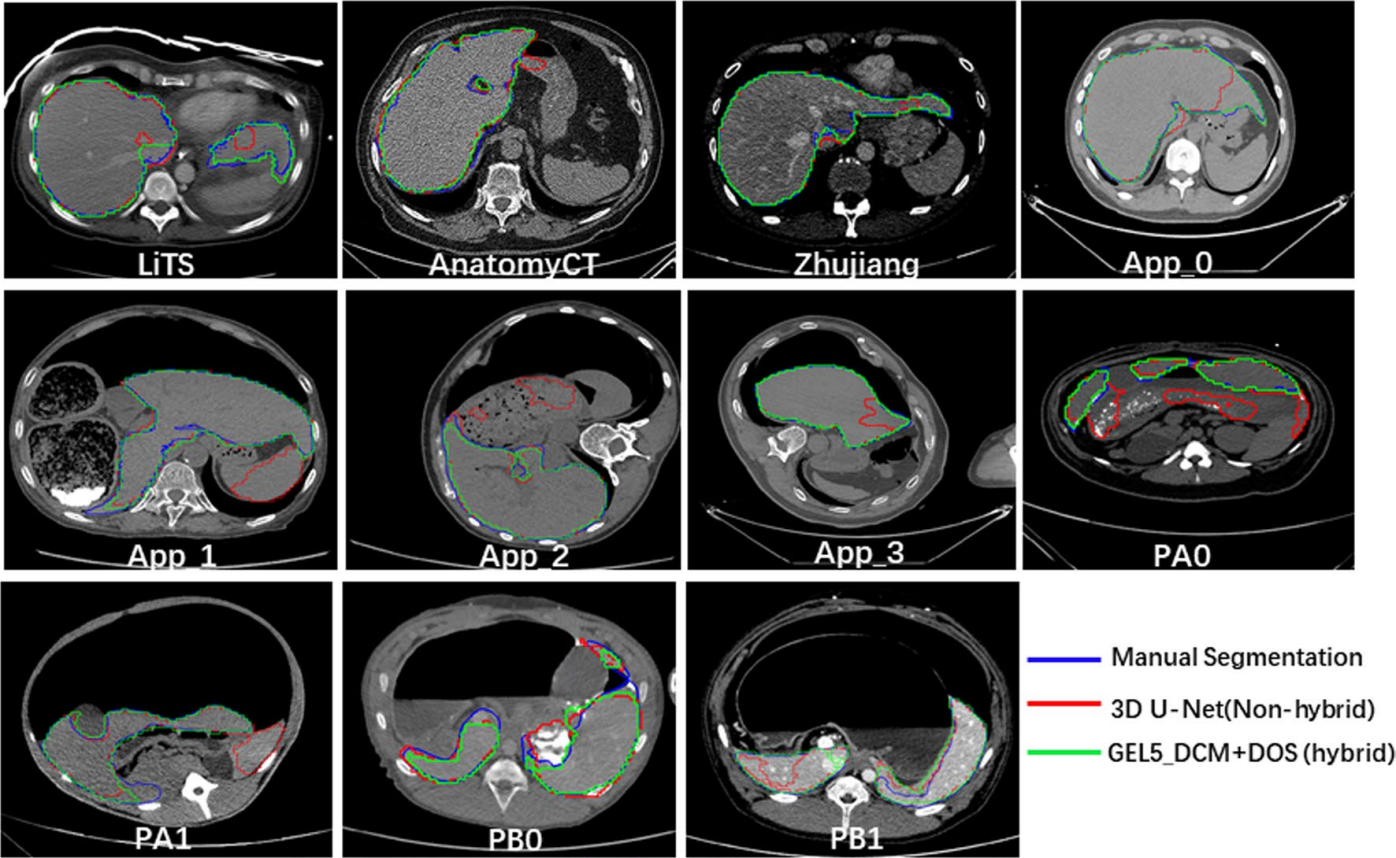
Visualization segmentation results of three comparison methods for hard examples by task. The blue, red and green line respectively show the segmentation results by reference segmentation, the simple 3D U-Net in two-fold non-hybrid training schema and the 3D U-Net in hybrid training with DOS sampling strategy and GEL_DCM layer sharing schema

Figure 6 further gives the visualization segmentation results of methods used in this paper by some hard samples for each task. Those samples have special liver shape with unclear boundary with nearby tissues such as spleen, which deduced less or over segmentation problem. It is obvious that the segmentation quality can be improved by using the hybrid training which means more plentiful training samples.

## Discussions

### Discussions about the pre-training from LiTS

When meeting the segmentation task of those datasets lacking any supervised labels, the first choice is exploiting related public dataset of the target organ segmentation. For the diverse liver CT datasets in our study, the most related is the largest and public liver CT dataset LiTS. We do experiments by pre-training from LiTS datasets and the results show that LiTS shows good generalization ability to human datasets but not porcine datasets.

**Fig. 7:**
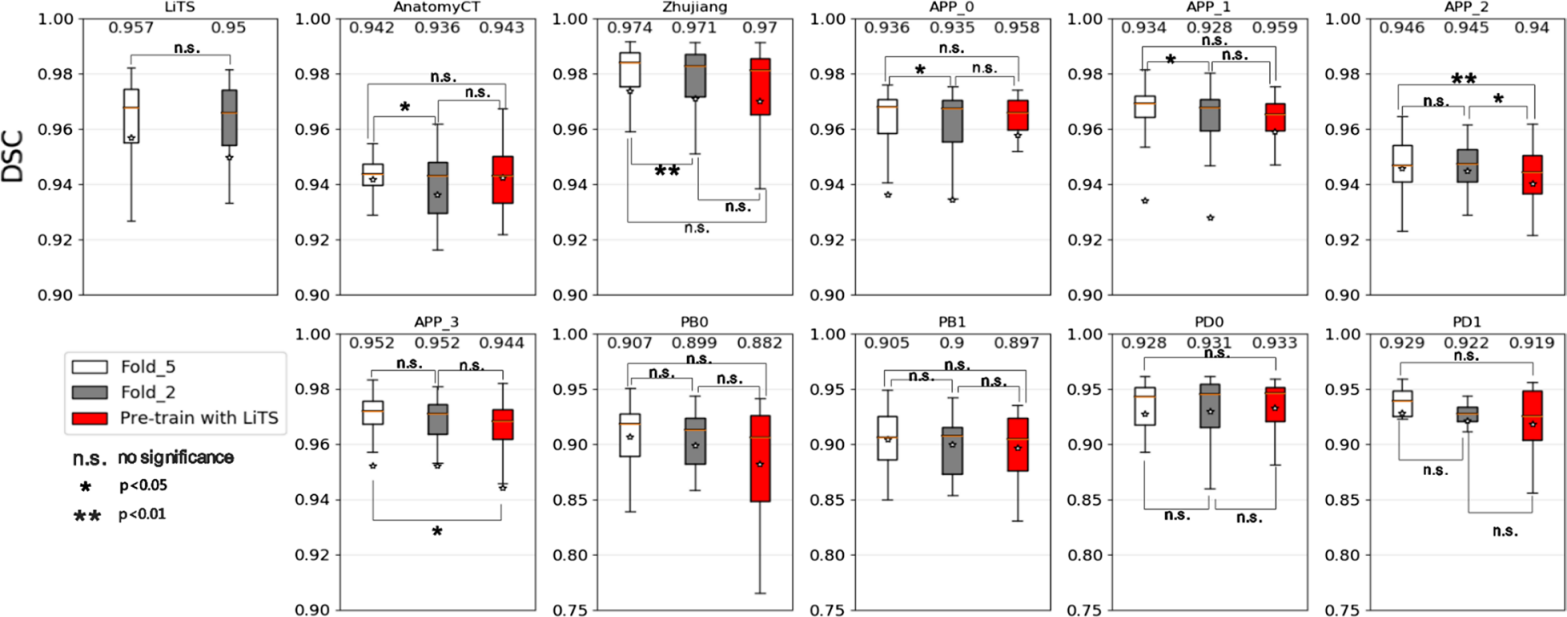
Box charts of comparison results measured with DSC for eleven datasets segmented by five-folds (white), two-fold (grey) and two-fold using LiTS Pre-trained model (red) respectively. For most datasets, there shows no great significance between the five-fold and two-fold results

In Fig. 7, we found that the impact of pre-trained model depends on its generalization performances relationship with the target dataset if the target task has enough training labels (i.e., 50%). The same trends can also be found in datasets like APP_2, APP_3 where the LiTS dataset has bad generalization ability in Table 2. The pre-trained model is effective for those datasets like Anatomy3, APP_0, and APP_1. The improvement for Anatomy3 was obvious in terms of average Dice value but insignificant in paired t-test. Because only one abnormal case in Anatomy3 contains a spleen larger than liver was easily taken as liver. By using the pre-trained model, such case can be segmented successfully. The APP_0 and APP_1 can be well predicted by the LiTS dataset and thus benefit from the pre-trained model. Tajbakhsh et al. [15] also found that using pre-trained model has no improvement when using all training samples. Besides, they have shown that the pre-trained model was very useful with small number of training samples. Our experiments also yield same conclusions. Recent study about the self-supervised pre-train model [16] shows better performances than fully-supervised pre-train model. This is the direction of our future study.

### Discussions about the compatibility of encoder levels

Related works such as Zeiler et al. [41] explored it by visualizing output feature maps of each level. They found that the first and second level convolutions learn very generative features like edges and colors. The third level learned more complex generative features such as textures. However, the fourth level learned discriminative features and the fifth level learned complete and most discriminative features. Shirokik et al. [43] measured the level generalization ability of a 2D U-Net segmentation model based on multiple MRI images from six domains. They compared three different models by fine-tuning the first, last and all levels. They concluded that first layers were more prone to domain shift than deeper levels. This is because those datasets differ in appearance such as intensities and were more generative in organ distributions. Then the low-level features contain more domain specific information. The multiple liver CT datasets in this study share more common low-level features such as texture and intensity features but differ in liver organ distributions due to different scanning profiles or the pneumoperitoneum pressure deduced large deformation and varieties of objects like patients and pigs from different populations. Then the high-level features encode the dissimilarities of those datasets. The Zhujiang dataset always achieved lower accuracy than fully supervised even with a separate encoder (GEL0_DCM). The compatibility may relate to the decoder.

One limitation of the study was the samples for each porcine dataset was too small due to the high cost in both economic and time. Besides, the image artifacts of the porcine datasets deduced by the leakage of artificial planned tumor show lower DSC value than real-patient datasets. The two factors may lead un-stable results for the porcine datasets as it may deduced completely different results when choosing another group of training samples. Another limitation of the study is that it did not relate to the few-shot learning as the few-shot learning was more useful for researchers when need to segment those special datasets from scratch, and this is the direction of our future study.

## Conclusion

In this paper, we used eleven liver CT datasets including both public dataset and dataset from previous clinical and animal experiment. Those datasets except the public dataset was completely new and thus have not been studied yet. We show their inter-dataset generalization ability relationships between porcine and patients, between images scanned with and without pneumoperitoneum pressure through cross-testing experiment. The results show that the Zhujiang were bad generalized to other datasets compared with public LiTS. The generalization between porcine and human was bad. The regularly-scanned dataset can well-generalized to irregularly-scanned dataset. Finally, we proposed a dataset-wise convolution module to prove that high-level encode more domain-specific information. The dataset unbalanced problem in hybrid training all datasets can be solved by setting DCM in the final level of the encoder. Moreover, the proposed DCM can be used in any level that encode the dissimilarities of multiple datasets.

## Data Availability

The datasets used or analyzed during the current study are available from the corresponding author on reasonable request.

## Abbreviations

CT: Computed tomography
DCM: Dataset-wise convolution module
MRI: Magnetic resonance imaging
SOTA: State of the art
GEL: Greater or equal to level
DOS: Dataset-order sampling
RSD: Random sampling from one dataset
RS: Random sampling
